# Person-centered care (PCC): the people’s perspective

**DOI:** 10.1093/intqhc/mzab052

**Published:** 2021-11-29

**Authors:** Gro Rosvold Berntsen, Sara Yaron, Morgan Chetty, Carolyn Canfield, Louis Ako-Egbe, Phuk Phan, Caitriona Curran, Isabela Castro

**Affiliations:** Norwegian Center for E-Health Research, University Hospital of North Norway, NSE, PB 35, Tromsø 9038, Norway; Institute of community medicine, UiT The Arctic University of Norway, Tromsø, UiT, PO Box 6050 Langnes, Tromsø N-9037, Norway; The Cochrane Collaboration St. Albans House, 57-59 Haymarket, London SW1Y 4QX, UK; Reach to recovery, Israeli Cancer Association, St Revivim 7, Givatayim 5348505, Israel; Patient for Patient Safety, World Health Organization, Avenue Appia 20, Geneva 1211, Switzerl; The International Society for Quality in Healthcare (ISQua), Huguenot House, 35-38 St. Stephens Green, Dublin 2 D02 NY63 IE, Ireland; Independent Practitioner Association Foundation (IPAF), 61 Juniper Road, Overport, Durban 4067, South Africa; Kwazulu-Natal Doctors Healthcare Coalition (KZNDHC), 61 Juniper Road, Overport, Durban 4067, South Africa; Department of Family Practice Faculty of Medicine, The University of British Columbia, Vancouver, 320 - 5950 University Blvd, British Columbia BC V6T 1Z3, Canada; The International Society for Quality in Healthcare (ISQua), Huguenot House, 35-38 St. Stephens Green, Dublin 2 D02 NY63 IE, Ireland; Health System Strengthening Cluster, WHO Country Office, One UN House, PAP, 2nd Street Sinkor, Monrovia, Montserrado 1000, Liberia; University Medical Center, 215 Hồng Bàng, phường 11, Quận 5, Ho Chi Minh, Vietnam; The International Society for Quality in Healthcare (ISQua), Huguenot House, 35-38 St. Stephens Green, Dublin 2 D02 NY63 IE, Ireland; Independent Practitioner Association Foundation (IPAF), 61 Juniper Road, Overport, Durban 4067, South Africa; Global Patient & Family Advisory Board, The Beryl Institute, 831 12th Avenue South, #212, Nashville, Tennessee TN 37203, USA; Planetree International, 130 Division St, Derby, Connecticut CT 06418, USA

**Keywords:** person-centered care, decision-making, shared, integrated care, proactive care

## Introduction

The call for person-centered care (PCC) is not new, yet despite a high priority over many decades and numerous frontline interventions, a lack of PCC persists [1]. We hypothesize that PCC will continue to be a secondary feature until PCC is a widely understood to be at the core of care quality.

## Why is PCC important?

We have witnessed enormous progress in biomedical care. Yet, both patients and health professionals have repeatedly voiced a concern that health-care systems (HCSs) do not sufficiently respect the individuality and human dignity of persons who seek their help. Even though the intertwined nature of person and body is well understood, in understanding a health challenge, the professional often comes to disregard identity and personhood. Ignoring the person in the patient is a profoundly troubling phenomenon. It undermines mutual understanding, empathy, trust and co-production and threatens PCC’s favorable clinical outcomes [2].

## What is PCC?

PCC is the art of embracing the patient as an equal partner in the design and co-production of care. PCC is a stepwise process following these concepts and principles:

1. HCSs’ goal is to improve and maintain ‘health’ understood as a resource for ‘what matters’ to the person in their context and life [3].
2. A patient journey (PJ) is the ensemble of care events organized by time across all diagnoses and providers to improve or maintain health for one patient. The PJ is the HCS core product [4].
3. There are three roles in every PJ: the patient, the professional(s) and a governance/payer, hereafter ‘the PJ partners.’
4. The governance/payer is an omnipresent third party, which shapes the PJ through design, funding and regulation of the HCS [5].
5. The principles of a high-quality PJ at the individual level are as follows:
  - Establish aim of PJ and concrete goals: a sensitive and empathic exploration of ‘what matters’ to the person [6], followed by a translation into relevant and realistic goals for care within professional, legal, ethical, and economic constraints set by governance/payer.
  - Co-production: PJ partners co-produce PJ goals, plans, delivery and evaluation of care, in alignment with ‘what matters’.
  - One person one plan: the professional(s) contribute condition-specific expertise and best practices across all conditions and help merge these into one care plan that serves PJ goals.
  - Proactive care: care plans build on the strengths of the patient, include self-care and self-management, anticipate needs and seek to prevent costly clinical crises in both human and economic terms.
  - Loyalty to plan: the PJ partners co-create care delivery according to the co-produced plan.
  - Evaluation, learning, and adjustment: the PJ partners evaluate care plan, delivery and goal attainment, as often as needed, in light of ‘what matters’ to learn and adjust the PJ.

## Why is it so hard?

Patients are persons who are already powerful in their lives. However, inherent features of health care contribute to disempowerment and distancing between patient and professionals, which results in incomplete professional knowledge of the person’s values, needs, preferences and context. The systematic focus on disease/condition/malfunction and professionally defined outcomes promote a paternalistic approach that may be distressing to the person [7]. Change relies on active identification of and counteraction against the depersonalizing side effects of professionalism.

## Sustainable and lasting system change

Frontline health-care professionals who deliver PCC often do so because it is the ‘right thing to do,’ not because it is a system feature. Change requires explicit system attention to PCC.

### Observe

Managing PCC means measuring and observing person centeredness. HCS must build patient-led evaluations of the PCC process at the individual and system levels, map disempowerment and de-personalization factors, complement measurements with user conversations and include those who belong to, or speak for, marginalized and vulnerable groups. These observations must be used actively in the plan for change.

### Plan and do

Reconfigure HCS so that regulatory, funding, organizational and information systems leverage PCC. Information systems should document, share and link ‘what matters’ to care decisions and delivery, goal attainment and clinical outcomes. Train for co-production at micro, meso, and macro levels and use economic and regulatory feedback to boost PCC achievements. Share the good stories. Research effective interventions, including effects on outcomes for patients, professionals and payers.

### Adjust

Continuously evaluate and measure progress, cycling between Observe-Plan-Do-Adjust, until patients’ reports of high-quality PCC become the norm [8].

## Conclusion—beacons of light

The current profession-centric HCS is built with the best of intentions but fails in terms of PCC. The paradigm change is already happening, as PCC emerges at the center of quality measurement [9] and care re-design [10]. In the new paradigm, care professionals are conscious of their role as “visitors” in the patient’s life. The patient is the host, guide and enabler of the healing journey. The goal is to enable the person to thrive in their life, with as little intervention from health care as possible.

## Data Availability

Not applicable.
